# Governance on the Drug Supply Chain via Gcoin Blockchain

**DOI:** 10.3390/ijerph15061055

**Published:** 2018-05-23

**Authors:** Jen-Hung Tseng, Yen-Chih Liao, Bin Chong, Shih-wei Liao

**Affiliations:** 1 Division of Risk Management, Taiwan Food and Drug Administration, No.161-2, Kunyang St, Nangang District, Taipei City 11561, Taiwan; 2 Department of Computer Science and Information Engineering, National Taiwan University, No. 1, Sec. 4, Roosevelt Rd., Taipei 10617, Taiwan; b03902103@ntu.edu.tw; 3 College of Chemistry and Molecular Engineering, Peking University, Beijing 100871, China; chongbin@pku.edu.cn

**Keywords:** blockchain, drug supply chains, Gcoin

## Abstract

As a trust machine, blockchain was recently introduced to the public to provide an immutable, consensus based and transparent system in the Fintech field. However, there are ongoing efforts to apply blockchain to other fields where trust and value are essential. In this paper, we suggest Gcoin blockchain as the base of the data flow of drugs to create transparent drug transaction data. Additionally, the regulation model of the drug supply chain could be altered from the inspection and examination only model to the surveillance net model, and every unit that is involved in the drug supply chain would be able to participate simultaneously to prevent counterfeit drugs and to protect public health, including patients.

## 1. Introduction

**Figure 1:**
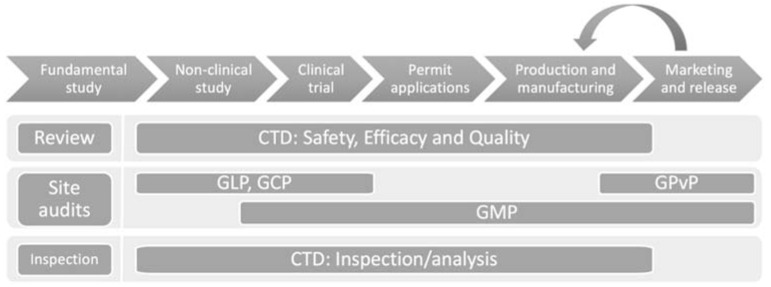
Medicinal product lifecycle management framework (CTD: Common Technical Document, GLP: Good Laboratory Practice, GCP: Good Clinical Practice, GPvP: Good Pharmacovigilance Practice, GMP: Good Manufacturing Practice).

The lifecycle of drugs, from development to the post market, includes basic research, non-clinical trials, clinical trials, licensing, manufacturing, and distributing/selling (Figure 1) [1]. Every stage of the life cycle requires good control and inspection to meet good practice.

On 6 September 2016, the Taiwanese government announced new regulations governing the tracking and tracing of medicinal products. These regulations are intended to control the sourcing and distribution of drugs. The new regulations cover three critical medical items: (1) plasma derivatives; (2) vaccines; and (3) botulinum toxin. They also cover twenty high-usage and high-priced drugs. These drugs must be included in tracking and tracing systems by 1 January 2018.

The purpose of the system is to reduce the risk of counterfeit drugs entering the legitimate supply chain, to quickly and effectively enforce the quality of drugs and to complete the recall of drugs as well as to protect the security and health of consumers.

When the drug supply chain is insufficient or too complex to surveil, one of the worst cases could be counterfeit drugs. The definition of counterfeit drugs can be defined by the World Health Organization (WHO), which states that “Products deliberately and fraudulently produced and/or mislabeled with respect to identity and/or source to make it appear to be a genuine product” [2].

It is difficult to measure the economic loss due to counterfeit drugs. The world does not even have accurate basic statistics, such as the number of counterfeit drugs. However, for the past few years, public opinion and experts have passively accepted the argument that 10% of medicines around the world could be counterfeit [3]. There are some suggestions to prevent counterfeit drugs. Some include: “improving management of supply chain”, “improving controls of secondary drug markets”, and “improving the use of technology to track and trace counterfeiting drugs” [4].

Among all the suggestions mentioned above, improving management of the drug supply chain is the most direct and holistic approach. From the procurement of drug ingredients, production, and distribution to the use of drugs, every step of the drug supply chain has an important role in drug safety. In this paper, we suggest introducing blockchain technology as a new tool or service platform to manage the drug supply chain.

## 2. Materials and Methods

### 2.1. Traditional Technologies Used in the Drug Supply Chain

With the emergence of mobile phones, mobile technologies including software and hardware to improve the track and trace of drugs in the supply chain are considered relatively mature and easy to adopt [5].

On the other hand, the application of the barcode scanning system or Radio Frequency Identification (RFID) technology in the drug supply chain is now relatively mature [6]. The characteristics of RFID, such as wireless and individual identification, make it possible to collect every step of information in the drug supply chain efficiently [7,8]. The surveillance of drug safety could be improved by introducing RFID technology in the drug supply chain, from anticounterfeiting, researching, storing, transporting, to selling [9].

### 2.2. Blockchain Technology Used in Public Health and Its Potential

Blockchain technology could create an encrypted, distributed, and immutable data ledger. In the public healthcare sector, previous researchers have concluded its usage to the sharing of information with stakeholders while ensuring data integrity and protecting patient privacy [10].

There have already been some applications using blockchain technology in healthcare management to create added value through medical data exchange and improving the privacy protection of patients [11]. Furthermore, some pilot researchers/startups showcased their design of the procedure to apply blockchain technology to healthcare and the drug supply chain, which includes IBM’s Watson Health artificial intelligence unit (Cambridge, MA, USA), iSolve LLC’s BlockRx^TM^ (East Norriton, PA, USA) and the Chronicled Company (San Francisco, CA, USA), etc.

When it comes to preventing counterfeit drugs in the drug supply chain, blockchain technology stands out as a way to ensure an immutable chain of transaction ledger, tracking each step of the supply chain at the individual drug level [12].

Although traditional technologies, such as mobile technology, barcode scanning system and RFID, have been suggested for the tracking and tracing of medicines, counterfeit-drug fiascos still happen on a worldwide scale. Even in relatively developed countries such as Taiwan, during March 2017, Crestor (rosuvastatin, 10 mg tablets) were found to be counterfeited by a similar ingredient called “Atorvastatin”. This event caused panic in the public, as some patients already had taken counterfeit drugs with lower efficacy for weeks [13].

Generally speaking, the government is expected to surveil every step of the entire drug supply chain to protect public health. Distrust is the most important reason the public needs the government as a supervisor. However, keeping every step under surveillance requires lots of resources and could be still inefficient. Therefore, blockchain technology is just one way to provide the ability to increase the efficiency of regulatory enforcement.

### 2.3. Gcoin Blockchain

#### 2.3.1. Characteristics of Gcoin Blockchain

Gcoin has been known for its governance design in the blockchain world. The letter G in Gcoin stands for Global Governance of the blockchain network. In this paper, we apply Gcoin blockchain’s double-spending prevention mechanism to alleviate the counterfeit-drug problem.

We use blockchain as an economical and mathematical-based way to establish trust between two or more parties who do not already know each other. There are lots of different ways in which this could be implemented. Each way of implementing blockchain brings along its own set of restrictions and benefits. We choose the Consortium Proof-of-Work approach in the Gcoin blockchain to address the double-spending problem or the anti-counterfeiting problem in the pharmaceutical world.

In Gcoin blockchain, different nodes of participants could be set to take different roles like coin issuers, full node, miners, or normal node in a hierarchy relationship.

#### 2.3.2. Identification of Drugs by Gcoin Blockchain

Gcoin blockchain can track every pill for drug identification, in a similar fashion to what blockchain accomplishes in Bitcoin. Every giver (seller) and receiver (buyer) in the drug supply chain has its own address (like the “address” in Bitcoin systems). For use in the drug supply chain, we suggest using batch or serial number, quantities, and all required drug information to generate a hash number as the public key. This public key could generate a Quick Response code (QR code) as the identification of the drugs (Drug ID).

#### 2.3.3. System Structure of Drug Transactions in the Supply Chain with the Gcoin Blockchain System

##### Participants and Their Roles

**Figure 2:**
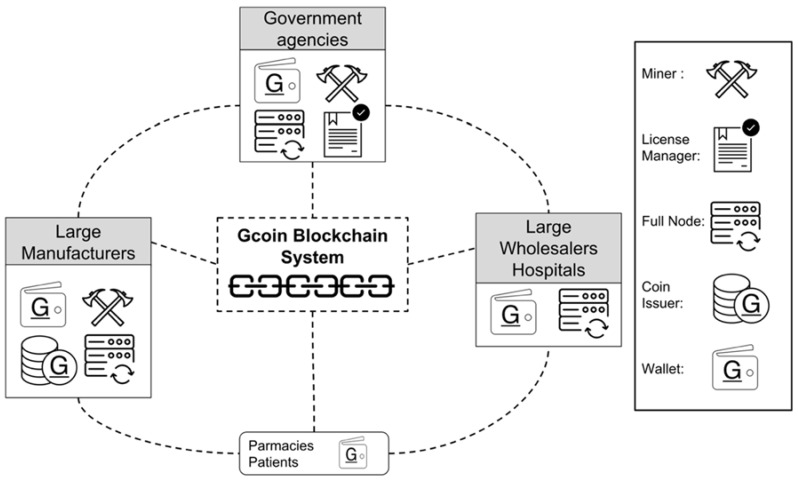
System structure and roles of participants.

Participants of drug supply chains include manufacturers, wholesalers, retailers, pharmacies, hospitals, and consumers (drug users). As for the hierarchy in the Gcoin system, the government authorities should surveil transactions and drug information and are recommended to take the role of an alliance member in the Gcoin blockchain system. An alliance member has the authority to issue a minter and miner license. Since the drug manufacturers are the source of drugs identified by the Gcoin blockchain system, they should take the role of coin issuer (minter). Miners who are in charge of verifying transactions and generating blocks are recommended to be large manufacturers and government agencies. The remaining large wholesalers, hospitals or third parties could be full nodes who are responsible for storing a backup of historical transactions. In addition, the remaining pharmacies and consumers should be the normal node (Wallet), which has the authority to implement transactions (Figure 2).

The personnel who are responsible for delivering, transacting, and inspecting the drugs in the drug supply chain would be issued the relative authority to sign identical digital signatures during their working procedure. We suggest using the Gcoin blockchain multisignature design, which is approved by successfully applying in the “NTU Help Center”.

#### 2.3.4. Workflow

**Figure 3:**
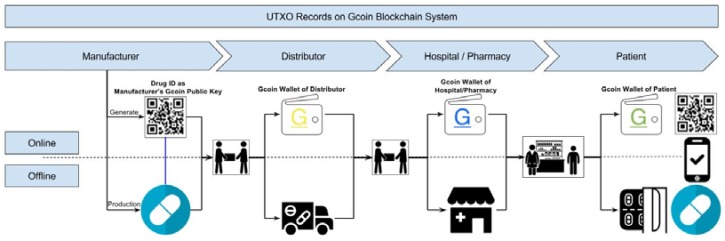
Workflow of the Gcoin blockchain system applying to drug supply chain.

As for a top to bottom drug supply chain (from drug manufacturers to consumers), manufacturers give their transaction data to drug receivers directly, and this data is recorded in the Gcoin blockchain. The transaction data of the digital signatures of the drug seller and buyer, the drug information (including the time stamp, location, item name, etc.), and the amount of drugs are verified on the chain. Then, all of this data is hashed as a digest to be recorded on the Gcoin blockchain (Figure 3). Whenever an illegal distributor wants to sell counterfeit drugs (with “fake” Drug ID mentioned above) to buyers, the transaction will be judged invalid because of the presence of fraudulent information about unspent transaction outputs (UTXO) stored in the Gcoin blockchain. On the other hand, unauthorized personnel are not able to carry out drug transactions in this system without a correct private key. Hence, the buyer/seller would be immediately aware of any anomalies within the transactions.

#### 2.3.5. The Performance of Gcoin Blockchain System

In our previous study, we proved that the Gcoin blockchain system was already able to be applied to financial fields, such as the automatic clearinghouse (ACH) business [14]. When conducting transactions in ACH business, the average speed could reach 17.5~26 transactions per second. Since the Gcoin blockchain system generates a block every 15 seconds, it could handle at least 1.51 million transactions in one day. According to the “2015 report of drug usage analysis” published by the National Health Insurance Administration in Taiwan, there are about 13.42 billion of drugs (3397 items) used in the health insurance system [15]. When considering the notice requirement of drugs, which should establish and declare track and trace records that are only less than 100 items, the processing ability of the Gcoin blockchain is more than sufficient.

### 2.4. Transparency: the Trend of Drug Supply Chains Regulation

#### 2.4.1. Trends in China

In China, according to the “Notice on the implementation of the opinions of “Two Invoice System” in the procurement of pharmaceuticals in public medical institutions (for trial)” and the “Regular press release in Jan. 2017” published by the National Health and Family Planning Commission, People’s Republic of China, the “Two Invoice System” will be implemented in the drug supply chain. In this system, drug manufacturers send drugs to distributors, and then to the hospitals with two steps. Only two invoices are legally permitted [16].

When receiving drugs, public medical institutions must verify the invoices, goods, and accounts at the same time. Not only must the invoice from the distributor be verified, but the invoice from the manufacturer should be verified as well [17].

#### 2.4.2. Trends in America

In America, the Drug Supply Chain Security Act (DSCSA) was enacted in 2013. The U.S. Food and Drug Administration has issued a series of guidance and policy documents. DSCSA requires interoperable systems to trace all pharmaceutical products in the U.S. Upon the request of an authorized body, all trading partners in the drug supply chain must share “transaction information” regarding the exchange of pharmaceutical products and must produce immediate transaction information and collect and produce all transaction information data originating from the drug manufacturer/repackager via an interoperable electronic system. In 2023, the DSCSA will implement a system to allow for the traceability of individual units of certain pharmaceuticals in the supply chain [18].

## 3. Results

In this paper, we suggest using the Gcoin blockchain system for improving the efficiency of information exchange in combination with an open government and decentralized autonomous organization (DAO) regulation model to make a secure and transparent drug supply ecosystem.

### 3.1. Drug Supply Chain Security Issues

The purpose of drug supply chains management is to increase efficiency and to benefit every participant through sharing the information of logistics and capitals to improve cooperation. According to McKinsey & Company, the cost of supply chain activities account for about 25% of the cost of the entire drug. As long as manufacturers, the government, and back-end medical institutions can improve cooperation, drug costs will be less [19].

When it comes to risk factors of the supply chain, there are several points mentioned by researchers from 2003 to 2013 summarized in a recent review. These risk factors include: “information infrastructure breakdown”, “information delays”, “lack of information transparency between logistics and marketing”, “lack of compatibility in IT platforms among supply chain partners”, and “Internet security” [20].

Many risk factors mentioned above could be mitigated by the Gcoin blockchain system and overall costs in the drug supply chain could be decreased because of the efficiency improvement of information exchange.

### 3.2. Surveillance Net Regulation Model

Since Gcoin blockchain can make the drug supply chain more trustworthy and transparent, government agencies could change the thinking of what and how to govern it. In this situation, a governance model of open government combined with the DAO model is the solution that we call the “Surveillance Net” regulation model.

#### 3.2.1. Open Government

The Organization for Economic Co-operation and Development (OECD) defines “open government” as “a culture of governance based on innovative and sustainable public policies and practices inspired by the principles of transparency, accountability, and participation that fosters democracy and inclusive growth” [21]. Open government is based on the transparency of government information, and the purpose of an open government is to ensure the accountability of government [22].

In an open government regulation model, open data is essential to achieve transparency and accountability. Hence this paper suggests making all of the drug supply chain transaction data protected by the Gcoin blockchain system open to all participants, including third parties, stakeholders, companies, and most importantly, all patients by their authority to view relative drug transaction data.

Using open data as a regulation tool has proven to be successful in America; for example, the “Toxics Release Inventory (TRI)” case. In the TRI case, the American government opened air pollution data to the public, and companies, upon realizing that their behavior was under surveillance by people, began to stop producing toxic substances. Thus eventually, transparency improves public health [23].

#### 3.2.2. Gcoin Blockchain System Smart Contract Applying to the Drug Supply Chain

“Smart Contract” is defined as a “computerized transaction protocol that executes the terms of a contract’’ [24]. In the Gcoin blockchain system, when the conditions meet the set conditions, the scripts as contracts will be triggered automatically by the distributed blockchain system. When it comes to the Drug Supply Chain, it is possible to set a serial of conditions on the Gcoin blockchain, such as the identities of sellers or buyers, medicine distribution amounts, or the auditing frequencies of medicine distributors and so on. With Gcoin blockchain, there is no central power that has the right to change a smart contract unless every full node on the Gcoin blockchain system comes to a consensus. Therefore, the Gcoin blockchain system makes DAO possible in the governance on the drug supply chains.

#### 3.2.3. Surveillance Net

With the Gcoin blockchain system, the transaction information of drugs is rather open but checked automatically through the drug supply chain. Additionally, by deploying a service layer upon Gcoin blockchain, we suggest designing smart contracts (programs such as those mentioned above that can execute automatically and operate DAO) in combination with an open government model. There are several approaches to increase surveillance efficiency. We provide two examples as follows:

Firstly, in the Gcoin blockchain system, every drug has only one identification and can only be sold once from one address (account) to another; this can prevent the double spending of drugs. In addition, every transaction is broadcasted to every participant to check if an anomalous transaction has occurred and will be stopped automatically according to the conditions set in the smart contract.

**Figure 4:**
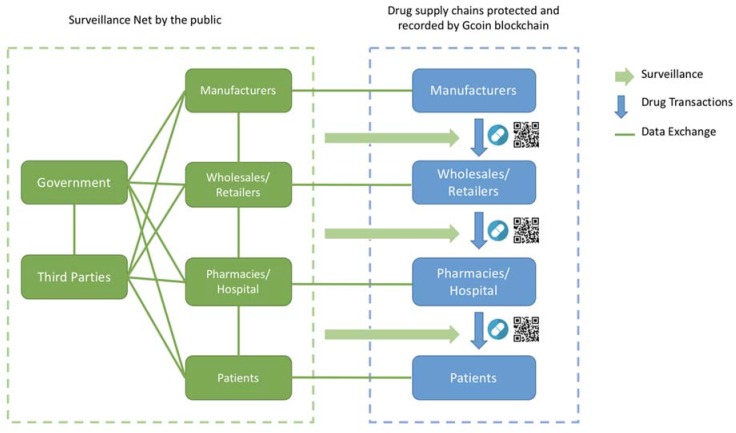
Surveillance net governance model of the drug supply chain.

Secondly, the government agencies could set a threshold or risk level by a transaction pattern discovered through data mining. If any transaction or the trading behavior of a company is not in compliance with the risk benchmark, the smart contract could trigger a warning message of inspection suggestion to the appropriate government agencies, and the potential illegal transaction will be judged invalid by the Gcoin blockchain system until the inspectors sent by competent authorities check the drugs and provide their digital signature to verify the transaction. The result of each inspection case is automatically fed back to revise the risk benchmark. The risk benchmark or inspection frequency could also be revised manually and periodically by every participant in the Gcoin blockchain system through a voting system to achieve the purpose of increasing participation and accountability (Figure 4).

With the Gcoin blockchain system, people including inspectors in the government have access to track and trace drugs in the supply chain without going into factories, warehouses, or pharmacies.

## 4. Discussion

Surveillance and inspection of the drug supply chain could be much more efficient and accessible because of transparency. Since blockchain is a new technology, laws and regulations about it are still under development. Even blockchain technology itself is under evolution (e.g., “smart contract”, blockchain as “Blockchain 2.0”), so further regulatory impact analysis and system simulation stress tests are needed in the future to conduct the cost-benefit analysis along with consultation to key stakeholders.

## 5. Conclusions

The drug supply chain transaction data established and protected by Gcoin blockchain is immutable, consensus driven, and transparent. With Gcoin blockchain, the governance model of the drug supply chain could shift from regulating (only by government audits) to surveillance net (by every participant who involves the supply chain).
